# A self-paced online emotion socialization intervention for parents of children with challenging behavior: *Tuning in to Kids OnLine*

**DOI:** 10.3389/fpsyg.2024.1393708

**Published:** 2024-08-29

**Authors:** Sophie S. Havighurst, Shaminka N. Mangelsdorf, Nikki Boswell, Jonathon Little, Abby Zhang, Kate Gleeson, Aniqa Hussain, Ann Harley, Alessandra Radovini, Christiane E. Kehoe

**Affiliations:** Mindful: Centre for Training and Research in Developmental Health, Department of Psychiatry, The University of Melbourne, Melbourne, VIC, Australia

**Keywords:** online, parenting, child, emotion socialization, intervention, behavior, *Tuning in to Kids*

## Abstract

**Background:**

Evidence-based parenting programs delivered using online technology are an important way to enhance program uptake. To date, programs that address emotion socialization processes, such as *Tuning in to Kids*, have always been delivered in person, via group or one-to-one delivery. This study used a randomized control design to examine the efficacy of the self-paced *Tuning in to Kids OnLine (TIKOL)*.

**Method:**

Participants were 150 parents of children aged 4-10 years old with challenging behaviors, randomized into intervention or 10-month waitlist control. Parents and teachers completed questionnaires at baseline and 6 months after the intervention (equivalent time points for controls) measuring parent wellbeing, parent emotion socialization, parent efficacy, child behavior, and anxiety.

**Results:**

Analyses, using mixed methods multilevel modeling, showed that intervention parents reported significantly reduced emotion dismissiveness and increased emotion coaching, empathy and efficacy compared to controls who did not. Parents participating in TIKOL also reported that their children's behavior problems and anxiety were significantly improved. Greater engagement (modules watched and duration of support calls) was associated with more significant improvements.

**Conclusion:**

Findings provide preliminary support for the efficacy of TIKOL in improving parents' emotion socialization and reducing child behavior problems and anxiety, especially when efforts to support online engagement are utilized. Further evaluation using independent observations and a sample representing a wider demographic would strengthen these findings.

**Clinical trial registration:**

Australian and New Zealand Clinical Trials Registry No. ACTRN12618000310268.

## Introduction

Emotion-focused parenting programs based on emotion socialization theory have been found effective in improving parenting and children's emotional competence (Havighurst et al., 2020). These programs are usually delivered either to groups of parents or via a professional in one-to-one sessions, however, as with many different parenting programs, barriers to access and engagement mean approximately only 10% of parents take up these opportunities, and only 60% of those will complete them (Thongseiratch et al., 2020). Barriers include structural and resource-based barriers, stigma associated with attending a parenting program and practical issues such as time constraints, limited transport, access, and inadequate childcare (Spoth and Redmond, 2000; Owens et al., 2002; Prinz and Sanders, 2007; Cuijpers et al., 2010). One way to address these barriers is through self-paced, online sessions (Metzler et al., 2012; Hansen et al., 2019). As more than 60% of the worldwide population, including socioeconomically disadvantaged populations, have internet access, web-based interventions can increase the accessibility of parenting interventions and are cost and resource efficient (Cuijpers et al., 2010; Cugelman et al., 2011; Metzler et al., 2012; Yap et al., 2018). Furthermore, examining program user engagement and uptake can inform ways to enhance engagement (Enebrink et al., 2012; Nieuwboer et al., 2013; Wetterborg et al., 2019). The current study examined whether an online version of the evidence-based, emotion socialization parenting program, *Tuning in to Kids* (TIK), was efficacious in reducing children's emotional and behavioral problems in a sample of parents who reported their child having challenging behaviors.

## Background

Parenting interventions can be delivered online as self-paced programs, with content delivered in pre-recorded free or pay-per-use materials containing psycho-education (e.g., about children's behavior; the role of parenting), video demonstrations (e.g., showing the intended parenting skills), activities that target specific skills (e.g., role plays or exercises to develop understanding about children's needs or practice ways of responding to the child), interactive quizzes or tasks, and additional supportive materials (e.g., resources, tip sheets etc.). They vary in length and may include additional elements of intervention support (phone calls or clinical consultation to boost application), and/or prompts to enhance engagement (e.g., automated reminders; phone calls).

The efficacy for self-paced online parenting interventions has been established with a variety of programs (Breitenstein et al., 2016; Sourander et al., 2016; Flujas-Contreras et al., 2019; Thongseiratch et al., 2020). A recent meta-analysis of 12 studies, with 2025 participants (2–12 years), examined the benefits of self-paced parenting interventions on emotional and behavioral difficulties in children (Thongseiratch et al., 2020). This study found the programs were similarly effective as face-to-face delivery, with a mean effect size of 0.32 in reducing children's behavior problems (Thongseiratch et al., 2020). An earlier systematic review of eleven experimental/quasi-experimental studies of self-paced online programs found a medium effect size (*d* = 0.61) for improvements in child behavior, and medium effect sizes (*d* = 0.46) for parent outcomes, including parental self-efficacy, wellbeing and parenting (Breitenstein et al., 2014).

Across reviews, programs were particularly effective when reminders were sent to parents and/or professional support was provided (Means et al., 2010; Richards and Richardson, 2012; Breitenstein et al., 2014; Baumel et al., 2016, 2017; Thongseiratch et al., 2020). Thongseiratch et al. (2020) found sending parents reminders regarding the program was the only individual factor used to enhance interventions that was effective in improving children's behavior problems. While adding phone calls to self-paced online programs has not been necessary for delivery (Thongseiratch et al., 2020), research is needed to understand which aspects of such calls aid program efficacy. Additionally, in evaluating a new intervention, phone calls can determine whether the intervention is received as intended.

The need to consider intervention components that enhance effectiveness and engagement, such as phone call support, is further evident in reviewing the outcomes of various iterations of Triple P Online (TPOL), an evidence-based self-paced online parenting program adapted from the widely used Triple P-Positive Parenting Program (De Graaf et al., 2008; Nowak and Heinrichs, 2008). In the initial iteration of TPOL, eight modules were used to deliver the intervention's core messages focused on development of parental self-efficacy, personal agency and self-sufficiency (Sanders et al., 2012). Additionally, several prompts were used to increase completion including: an email reminder to start, a 5-minute re-engagement phone call and email if the participant had not logged on for 3 weeks, and phone contact (average of 11 min per call) at 2 and 5 weeks following commencement to address any technical problems (no clinical support provided). In an evaluation of this original version of TPOL, all modules were completed by 47% of participants by 6 months, who took an average of 5.9 h to complete the program (Sanders et al., 2012). Results from this trial showed improvements in child behavior, dysfunctional parenting, parental confidence and anger (Sanders et al., 2012). Interestingly, when Day and Sanders (2018) compared the delivery of TPOL with and without a phone support call in a sample of parents (*n* = 183) of children 1–8 years with concerns about behavior, it was evident that the inclusion of clinical support calls (rather than simply phone contact to prompt engagement) significantly enhanced outcomes (Day and Sanders, 2018). They found that 94% of all parent and child outcomes in the telephone-supported TPOL condition (up to eight clinical calls intended to review content, practice tasks and set goals) were significantly better than waitlist controls at 5-month follow-up, while only 50% of outcomes were significantly better than waitlist controls for the TPOL group without clinical phone support (Day and Sanders, 2018). Further to this, there was notably higher program adherence (53.0% with phone support, 22.8% without phone support) and more modules completed (Mean = 5.62 and 3.25, respectively) in the phone call-supported TPOL group (Day and Sanders, 2018). A positive correlation was also seen between the number of support phone calls and the number of online modules completed, although phone call support and other elements of intervention engagement and adherence were not directly analyzed as predictors of intervention outcomes.

While the literature indicates that, overall, self-paced online parenting programs can be effective, especially if parent engagement is enhanced through support calls, very few programs have focused on enhancing emotional outcomes for parents and children (Thongseiratch et al., 2020). Exceptions are Morgan et al. (2017), who successfully aimed to improve child anxiety using behavioral parenting techniques such as graded exposure, contingency management, and use of rewards to help children better manage anxiety; and Jones et al. (2017), whose program modules focused on supporting parents diagnosed with bipolar disorder to manage their own emotions in order to address child behavior problems. Given these preliminary findings, and the significance of emotion-related parenting for optimal development in children, investigating if emotion-focused online programs enhance these factors is important (see Scott and Dadds, 2009).

Emotion-focused programs are based on emotion socialization (ES) theory and have been found efficacious in improving parenting and reducing children's behavior problems (see Havighurst et al., 2020 for a review). Children learn about emotions by observing their parents' emotion expression, via parents' responses or reactions to their emotions (parents can either encourage or dismiss emotions), through direct teaching about emotions in discussion (including emotion coaching), and via the emotional climate of the family (Gottman et al., 1996; Eisenberg et al., 1998). Parents' beliefs about emotions, or Meta-Emotion Philosophy (Gottman et al., 1996), are primarily established during childhood through family of origin and cultural experiences, and impact their parenting ES practices.

*Tuning in to Kids* (TIK: Havighurst et al., 2010, 2015) is an emotion-focused intervention that helps parents develop skills in understanding and regulating their emotions, explore the origins of their Meta-emotion beliefs and their impact on parenting, and learn skills to alter automatic reactions to their children's emotions that may be dismissive or disapproving of emotions. Additionally, the program focuses on helping parents to use the five steps of Emotion coaching that include noticing the child's emotions, connecting with them when they occur, empathizing with the child's experience, naming the emotion, and if needed, providing support to solve problems or set limits around behaviors (Gottman and DeClaire, 1997). This, in turn, facilitates parent-child connection and promotes parents' efficacy in their parenting role. There is now considerable evidence of the effectiveness of group and one-to-one delivery of TIK in community (Havighurst et al., 2004, 2010; Wilson et al., 2012) and clinical samples (Havighurst et al., 2013, 2015, 2021; Mastromanno et al., 2021). Effect sizes for changes in parent emotion socialization are often medium to large; with change in children's emotional competence and behavior being small to medium, and larger effects in clinical samples. TIK has now been disseminated in many different countries (e.g., Aghaie Meybodi et al., 2019; Edrissi et al., 2019; Bølstad et al., 2021; Chan et al., 2021; Burkhardt et al., 2024), with programs offered in a range of service settings, and evaluations showing similar effectiveness to the original evaluations of TIK (see also Havighurst et al., 2020, 2022). However, with delivery of the program occurring primarily in face-to-face settings, as with other parenting programs (Hansen et al., 2019), real-world uptake remains relatively low. Thus, TIK was adapted for online, self-paced delivery.

The *Tuning in to Kids OnLine* (TIKOL) program was adapted by the original TIK creators (Havighurst and Harley), TIK team members and a film production team. Video materials of parent-child interactions were created by an Aboriginal and Torres Strait Islander film company (Sista Girl Productions) to increase acceptability for a culturally diverse audience. Program content is delivered by the first author with footage of parent-child interactions overlaying dialogue to enhance engagement and demonstrate concepts. TIKOL is a much shorter time commitment of 130 min vs. either 720 min (community delivery) or 960 min (clinical/high risk delivery) in a TIK face-to-face group/one-to-one delivery. Nevertheless, the ten TIKOL modules (5–15 min each) include all core content from the original TIK program and only exclude the interactive elements of group/one-to-one delivery (i.e., discussion, dyadic exercises and role plays). Consistent with the TIK manualized delivery, learning is scaffolded across the modules via specific activities aimed to enhance parent emotional competence and skills in emotion coaching. In the original TIK program the use of role plays has been viewed as integral for parents. Role plays allow observation and practice of emotion coaching skills and offer parents opportunities to see this response style in contrast with an emotionally dismissive response. In TIKOL, videos of different mother/father—child interactions that contrast emotion dismissing and emotion coaching using actors were provided in each module, including commentary detailing the key teaching points. To provide personal stories of parents' experiences with TIK, interviews with parents who had participated in TIK group programs were filmed and interspersed, outlining some of the challenges in learning the emotion coaching skills. Finally, parents were provided with opportunities for reflection and encouraged to practice skills between modules.

In addition to the program modules, because parents usually find the emotion coaching skills hard to apply, two phone calls were offered to each participant to trouble-shoot any difficulties with the material and step through at least one example of when they had attempted to use emotion coaching. This often resulted in validation of the parent's experience in learning the skills, guidance in how to apply the skills accurately and support for the parent with their own emotional wellbeing.

## Study aims

The current study evaluated TIKOL in a randomized controlled trial with parents of children with challenging behaviors. The first aim was to examine efficacy by comparing those receiving the intervention with those in a control condition on different outcomes. The research questions were:

- Does TIKOL lead to improved parent emotion socialization (decreased parent emotion regulation difficulties, decreased emotion dismissiveness and increased emotion coaching and empathy)?

- Does TIKOL lead to improved child internalizing and externalizing behavior?

A second aim was to explore if program engagement, measured via module completion and phone support calls, enhanced program outcomes. The research question was:

- Does greater program engagement (percentage of modules watched and duration of support calls) predict improvement on parent and child outcomes?

## Method

### Participants

Participants were recruited from March 2018 – July 2020, and were 151 primary caregivers (91% mothers; *M*_*age*_ = 40.7 years; *SD* = 4.6), who self-identified as having a child aged 4–10 years with challenging behaviors (*M*_age_ = 6.6 years; *SD* = 1.8; Range). The majority of families consisted of biological parents living together (80.8%), with an average household income of $100,000 or more (68.9%). Gross family income ranged from low to high. The average gross household income in Australia is $116,654 (Australian Bureau of Statistics, 2019). Most participants were in the workforce, either part-time (56.3%) or full-time (21.9%). They predominantly spoke English as a first language (79.5%), nearly all had completed high school (97.4%) and all had a post-school qualification with the highest percentage having completed a bachelor's degree (43.7%), post-graduate degree (31.1%), or graduate diploma/certificate/Advanced diploma/diploma (23.9%). For children in the study, 62.9% were above the clinical cut-off for intensity of problematic behaviors [>130 on the ECBI intensity subscale, *M*_(151)_ = 0.629, *SD* = 0.484]. Child anxiety was generally not in the clinical range except 2% had Physical Injury Fears. Demographic characteristics of participants can be seen in Table 1.

**Table 1:** Participant demographic characteristics.

		**TIKOL (78)**	**Control (73)**
Child	Child gender (% of boys)	60.30%	69.90%
	Age in years (SD)	6.7 (1.8)	6.46 (1.8)
Family	**Relationship status (%)**		
	Single parent	12.80%	6.80%
	Blended family	0.00%	1.40%
	Biological parents living together	78.20%	83.60%
	Biological parents not living together	1.30%	2.70%
	Other	7.70%	5.50%
	**Income (%)**		
	$0–19,999	2.60%	2.70%
	$20,000–39,999	2.60%	5.50%
	$40,000–59,999	5.10%	5.50%
	$60,000–99,999	19.20%	19.20%
	$100,000–180,000	38.50%	35.60%
	$180,000–or more	32.10%	31.50%
Parent	Gender (% mothers)	88.50%	91.80%
	Age in years (*SD*)	40.89	40.4
	English as first language (%)	78.20%	80.80%
	**Work hours (%)**		
	Full time	28.20%	15.10%
	Part-time	51.30%	61.60%
	Unemployed	6.40%	8.20%
	Not in work force	14.10%	15.10%
	Completed high school (%)	97.40%	97.30%
	**Post school qualification (%)**		
	Bachelor degree	42.30%	45.20%
	Postgraduate degree	33.30%	28.80%
	Graduate diploma/certificate	11.50%	6.80%
	Advanced diploma/diploma	6.40%	8.20%
	Certificate	6.40%	8.20%
	Other	0.00%	2.70%
	Mental health issues in the past year (% yes)	30.80%	32.90%

Bolded values are significant at <.05.

### Procedure

This study was part of a larger four-armed randomized control trial (*N* = 307) investigating the efficacy of different methods of intervention delivery of the Tuning in to Kids parenting program (i.e., one-to-one, group, online, and waitlist control). Participants were recruited via advertisements (i.e., information letters and cards) in childcare centers, kindergartens, schools, medical centers and on social media. Expressions of interest were welcomed from parents identifying their child as having “challenging behavior” (rather than behavior problems) that could include internalizing or externalizing behaviors. This was because we wished to identify children at risk for a range of mental health difficulties. Interested parents contacted the research team directly and their eligibility was assessed. Inclusion criteria were being the parent of a child aged between four and ten who the parent perceived as presenting with challenging behaviors. Only one parent and one target child from any given family was able to enroll. Parents were excluded if they indicated to the research team that they did not have sufficient English to participate in the assessment or the intervention (and alternative support service referrals were made), or if their target child had a diagnosis of a severe Communication or Pervasive Developmental Disorder.

**Figure 1:**
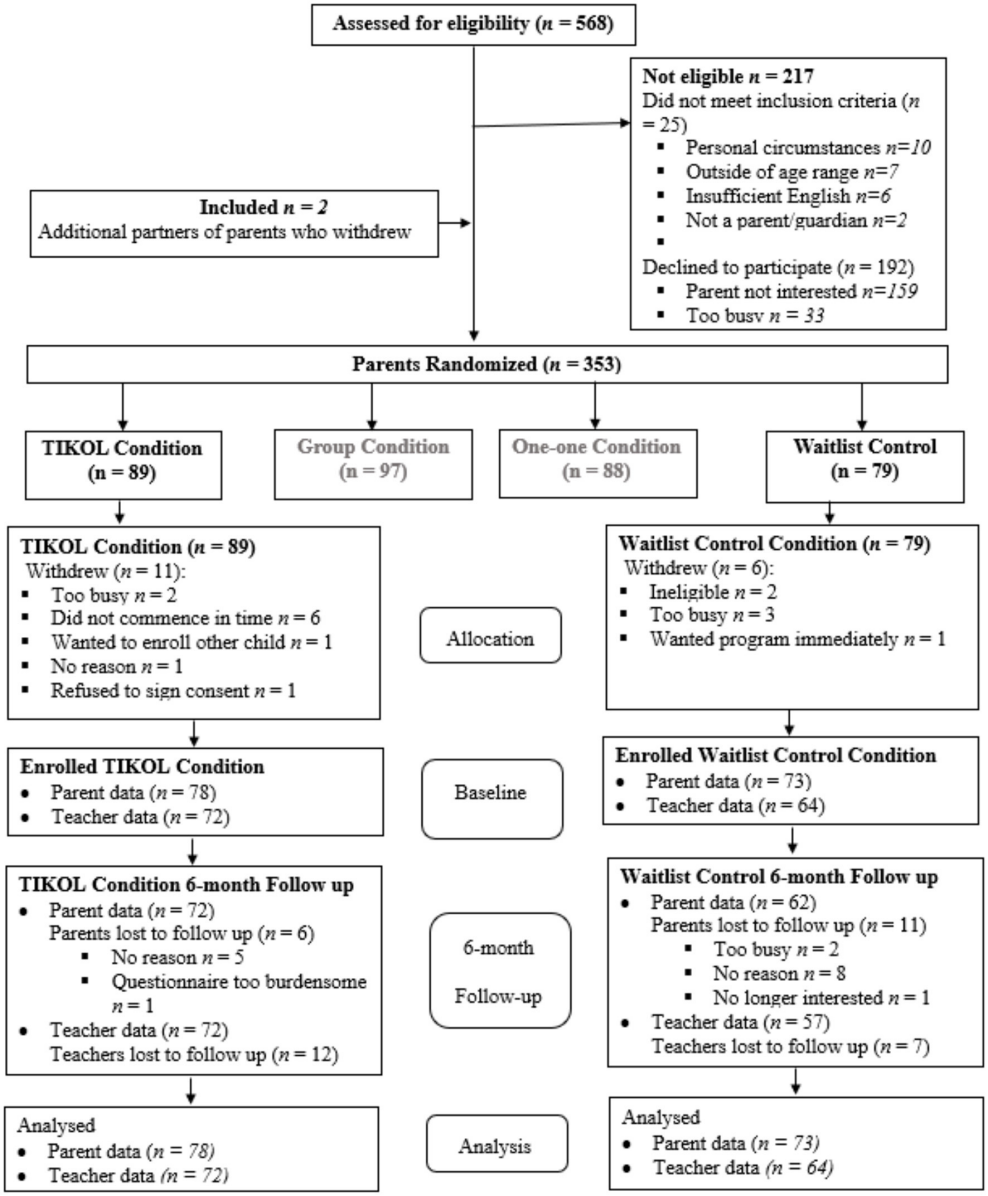
Participant flowchart.

Eligible parents were provided with information via phone or email, and if interested, were emailed a consent form and link to a Qualtrics questionnaire. Parents provided contact details for their target child's teacher and consent for the research team to contact the teacher to complete a Qualtrics questionnaire. Following baseline questionnaire completion by parents and teachers, parents (*n* = 353) were randomized [using the Research Randomizer software, in blocks of 20 (Urbaniak and Plous, 2013)] to one of the four study arms. The current study reports on the outcomes for the TIKOL vs. control arms of the study only (*n* = 168). Parents assigned to TIKOL (*n* = 89) were sent a link to watch the program on Qualtrics. Parents assigned to the control condition (*n* = 79) were told that they would receive their link to the online program in 9 months' time. Of the 168 participants randomly assigned to either TIKOL or control (see Figure 1), 17 participants (TIKOL *n* = 11; WL control *n* = 6) withdrew after completion of the first questionnaire, leaving a final sample of 151 (TIKOL *n* = 78; WL control *n* = 73). Participants who withdrew after randomization were less likely to have completed high school (88.2% completion) when compared with the remaining baseline sample [97.4% completion; χ^2^(2) = 7.352; *p* = 0.025]. No other differences were found. Reasons for withdrawal were primarily being “too busy” (see Figure 1). Of the 151 parents in the study, 17 (six intervention, 11 control) failed to return questionnaires at 6-month follow-up (11%). There was no significant difference between parents failing to return questionnaires at follow up (*n* = 17) and the rest of the sample (*n* = 134; *p* > 0.05) on any of the measures. Children's teachers completed baseline (*n* = 136) and follow up questionnaires (*n* = 117) with the majority being new teachers at follow up (*n* = 95) due to children moving to a new school year. Two independent sample *t*-tests were conducted with baseline parent-reported child outcomes (child anxiety and behavior problems) between participants whose teachers returned or withdrew questionnaires at baseline (responded *n* = 136, withdrew *n* = 15) or follow up (responded *n* = 117, withdrew *n* = 34). No significant differences were found in either of the analyses (*p* > 0.05).

To enhance program engagement and understanding, TIKOL parents were offered two support calls 3 weeks after receiving the Qualtrics link and once they had progressed to the later modules. Of the 78 intervention parents, 70 (90%) completed the first call, and 57 (73%) completed the second call. Eight parents (10%) did not respond to either call. The first call ranged from 8 to 70 min (*M* = 29 min, *SD* = 14 min) and the second ranged from 4 to 96 min (*M* = 29 min, *SD* = 16 min). Parents were encouraged to watch the program within an 8 week period, and took an average of 8 weeks to watch 10 program modules [*M*_(78)_ = 54 (days), SD = 46 (days); with the average time taken to start watching the modules being 2 months [*M*_(78)_ = 54 days, *SD* = 46 days]. The percentage of parents in TIKOL who watched at least 60, 80, or 100% of the modules was 78.1, 72.6, and 33%, respectively, with an average module completion of 81% (*SD* = 27.87%). Follow up data was collected 9 months after baseline [*M*_(78)_ = 240 days, *SD* = 71 days] to give parents sufficient time to apply skills learnt in TIKOL. For the control condition, follow-up data was collected 9 months after baseline to be equivalent to those in the TIKOL condition. Follow-up data were obtained from 72% of TIKOL and 62% of control participants.

The study was designed to adhere with the CONSORT guidelines (Schulz et al., 2010). Approval was obtained from The University of Melbourne, the Department of Education and Training, and the Catholic Education Office. The study was registered with the Australian and New Zealand Clinical Trials Registry (ACTRN12618000310268).

### The intervention: *Tuning in to Kids OnLine*

TIKOL is a 130-min self-paced version of the *Tuning in to Kids*^®^ parenting program. The theoretical basis draws from emotion socialization, as well as attachment, mindfulness and neurobiological theories (see Havighurst et al., 2021). TIKOL teaches parents/carers to use the five steps of emotion coaching outlined by Gottman and DeClaire (1997). Activities in the program were developed to increase parent awareness, understanding and regulation of their own emotions (exploring family of origin experiences, their attitudes toward emotions and learning skills in self-care and emotion regulation); perspective taking and empathy; and skills in assisting children to regulate emotions. The program is presented in 10 modules providing psychoeducation, exercises, parent-child videos demonstrating emotion dismissing and emotion coaching, discussion about the videos, and parents sharing their experience of the program. Participants were sent a link to TIKOL, which included advice about how to work through the program, downloadable materials with extra information and exercises, and the video modules. The intervention included two support calls to aid program implementation and to answer questions. Support calls were made by the project manager (last author), a clinical psychologist working as senior research assistant on the study (second author), and students enrolled in a counseling master's degree (*n* = 1), or PhD (*n* = 2). The content of the calls was shaped by structured questions, including: (1) Have you been able to access the program modules and if so, how many modules have you watched? (2) Are you having any technical difficulties accessing the modules? (3) How are you finding the program and do you have any questions regarding the content or about trying out the skills with your child? and, (4) Do you have an example of when you have used emotion coaching with your child?

### Measures

#### Parent measures

The *Difficulties in Emotion Regulation Scale* (DERS-16; Bjureberg et al., 2016) was used to measure difficulties in parental emotion regulation. Items are rated on a 5-point Likert scale, ranging from 1 (Almost never) to 5 (Almost always). Higher scores represent higher levels of difficulties. The DERS-16 and the DERS-36 have shown reliability above 0.80 for all subscales, and total Cronbach's alpha of 0.94 and 0.95, respectively (Hallion et al., 2018). In the present study using the DERS-16, Cronbach's alphas at baseline and follow-up were 0.93 and 0.94, respectively.

A subscale from the *Depression, Anxiety and Stress Scale* (DASS-21 items; Lovibond and Lovibond, 1995) was used to measure parent anxiety over the past week. Specifically, the anxiety subscale (example item: “*I was worried about situations in which I might panic and make a fool of myself* ”) rates parental self-reported anxiety on seven items (rated on a 4-point scale from 0 = “did not apply to me at all” to 3 = “applied to me very much or most of the time”), with higher scores indicating greater anxiety. This subscale is widely used and has shown good internal consistency. Cronbach's alphas at baseline and follow-up in the current study were acceptable at 0.70 and 0.76 for at baseline and follow-up, respectively.

The *Parent Emotional Style Questionnaire* (PESQ), was used to assess parental beliefs about their child's emotions. This instrument was adapted from the 14-item Maternal Emotional Style Questionnaire (MESQ; Lagacé-Séguin and Coplan, 2005), by Havighurst et al. (2010) to include seven additional items capturing parents beliefs about children's fears and worries. Within the PESQ, the Emotion Coaching subscale includes 11 items (e.g., *anger is an emotion worth exploring*), and the Emotion Dismissing subscale includes 10 items (e.g., *childhood is a happy-go-lucky time, not a time for feeling sad or angry*), rated using a 5-point Likert scale (Ratings range from 1, *strongly disagree* to 5, *strongly agree*). Havighurst et al. (2010) also selected the five PESQ items that tapped parents' empathy and emotional connection with their child (e.g., *when my child is scared, it's an opportunity for getting close; when my child is angry, I take some time to try to experience this feeling with him/her*) to create a subscale of Empathy. In previous research the 21 item PESQ has shown acceptable to good reliability with a Cronbach's alpha ranging from 0.68 to 0.83 for Emotion Coaching and 0.81–0.82 for Emotion Dismissing (Wilson et al., 2014). In the current study, all sub-scales showed acceptable/good reliability at baseline (Emotional Coaching Cronbach's alpha 0.78; Emotion Dismissing Cronbach's alpha 0.85; Empathy Cronbach's alpha 0.76) and at follow up (Emotional Coaching Cronbach's alpha 0.76; Emotion Dismissing Cronbach's alpha 0.88; Empathy Cronbach's alpha 0.75).

The *Coping with Children's Negative Emotions* (CCNES; Fabes et al., 1990) was used to assess how parents respond when their child experiences negative emotions. Parents answer questions relating to how they might react to their child in twelve different scenarios (e.g., *If my child loses some prized possession and reacts with tears, I would…*), on a 7-point Likert scale (Rating from 1, *Very unlikely* to 7, *Very likely*). The CCNES contains 7 subscales, each measuring a type of response to a child's emotions; *Expressive Encouragement, Emotion-Focused Reactions, Problem-Focused Reactions, Punitive Reactions, Minimization Reactions, Distress Reactions*, and *Acknowledging Emotions*. The *Acknowledging Emotions* subscale was added by the research team to capture empathy/acknowledgment of emotions, a key component of the emotion coaching construct and one of the 5-steps outlined by Gottman and DeClaire (1997). For all subscales, higher scores represent higher levels of that construct. The Expressive Encouragement, Acknowledging Emotions and Problem Focused Reactions subscales were combined to represent an overall Emotion Coaching subscale. The Minimization and Punitive subscales were combined to represent an overall Emotion Dismissing subscale. Subscales of Emotion-focused reactions and Distress Reactions were omitted due to low reliability. The CCNES has acceptable to good reliability and validity with Cronbach's alpha ranging from 0.69 to 0.85 across sub-scales (Fabes et al., 2002). In the current study, Cronbach's alpha showed good to excellent reliability on all scales at baseline (ranging from 0.87 to 0.94) and follow up (ranging from 0.88 to 0.93).

The *Parental Sense of Competence Scale* (PSOC; Gibaud-Wallston and Wandersman, 1978) was used to measure parental efficacy (the degree to which a parent feels competent and confident in handling child problems) and satisfaction (the quality of affect associated with parenting). The 17 items scale includes items such as, “My mother/father was better prepared to be a good mother/father than I am.” Responses are rated on a 6-point Likert scale (Ranging from 1, *Strongly disagree* to 6, *Strongly agree*), with some items reverse coded. Higher scores indicate a higher parenting sense of competence. Previous research found good reliability for the measure with Cronbach's alpha ranging from 0.77 to 0.82 (Rogers and Matthews, 2004). In the current study the efficacy subscale was used with Cronbach's alpha was 0.81 at baseline and 0.91 at follow up.

#### Child measures

The *Eyberg Child Behavior Inventory* (ECBI; Eyberg and Pincus, 1999) was used to measure child behavior, which contains 36 behavior problem items e.g., “Acts defiant when told to do something”. Parents rate each item on a 7-point Likert scale from 1 (Never) to 7 (Always) to indicate how often this is a problem for them, additionally they tick yes/no if the behavior is a current problem. Two separate scores are calculated; an *Intensity score* using the sum of all items and the *Problem score* as the sum of the number of times the parent indicated “yes” to an item being a problem. Higher scores represent higher levels of behavior problems. Cut off scores of 130 for the *Intensity* scale and 11 for the *Problem* scale are used to identify clinically significant behavior problems. Previous TIK research studies have found excellent reliability with the ECBI (Cronbach's alpha 0.90 to 0.93) (Havighurst et al., 2010). In the current study, Cronbach's alphas were excellent at baseline and follow up (0.91 and 0.94, respectively, for the Intensity score and 0.88 and 0.90 for the Problem score).

The *Spence Child Anxiety Scale-Parent version* (SCAS; Nauta et al., 2004) was used to measure child anxiety. This 39 items scale (two items were removed for the purposes of this study as they did not apply to children of younger ages—being home alone and taking tests) includes items such as, “My child is scared of the dark”. This measure has demonstrated strong psychometric properties for assessing childhood anxiety in community and clinical samples (Whiteside and Brown, 2008; Arendt et al., 2014). The SCAS parent version measures six subscales, each related to a different form of anxiety: Panic Attack and Agoraphobia, Separation Anxiety, Physical Injury Fears, Social Phobia, Obsessive Compulsive and Generalized Anxiety Disorder. High internal reliability (Cronbach's alpha 0.87 to 0.94) has been reported in clinical and community samples (Ramme, 2008). A total anxiety score was used in the current study with higher scores represent higher levels of anxiety. Cronbach's alphas at baseline and follow up were each 0.90.

The *Sutter-Eyberg Student Behavior Inventory—Revised* (SESBI-R; Eyberg and Pincus, 1999) was used to measure teacher-reported child behavior problems. This is a 38-item measure assesses children's behavior problems at school. Teachers were blinded to children's intervention condition. Items relevant to the child's behavior (e.g., “has temper tantrums”) are rated on a 7-point scale from 1 (never) to 7 (always) to generate an intensity score. Intensity scores were used in this study, with scores above 150 indicating clinically significant behavior problems. This is a commonly administered and well-validated measure of teacher-reported behavior problems that often has reported high reliability of over 0.90 (Querido and Eyberg, 2003). In the current study, the Cronbach's alpha at baseline was 0.98 and 0.96 at follow up.

### Analytic strategy

Regression analyses using SPSS Mixed Models were used to investigate improvements in both parent and child outcomes from baseline to follow-up (i.e., 9 months post baseline). First, baseline and follow up data were examined for missing values, normality and outliers. There were minimal (<6%) missing values at baseline, and Little's missing completely at random test was not significant, $x_{\left(281\right)}^{2}$ = 285.527, *p* = 0.414, indicating that all variables used within the SPSS MIXED model analyses were missing completely at random (MCAR; Little, 1988). Therefore, missing data were treated using the SPSS MIXED maximum likelihood procedure with restricted maximum likelihood (REML), robust when data meets Rubin's (1987) missing-at-random (MAR) assumption. When estimating population parameters the SPSS Mixed procedure only uses information contained within the dependent variable and is less inclusive than strategies that use all available information. As a second step, to account for missing follow-up data and to improve the plausibility of the MAR assumption, a more inclusive strategy was used which allows for all cases to be preserved in line with an intent-to-treat (ITT) analysis. This strategy used all 16 study variables (not just the dependent variable) with Mplus 8.10 (Muthén and Muthén, 1998–2017) to generate 100 multiple imputed datasets using a Markov chain Monte Carlo (MCMC) algorithm. The multiple imputation was done separately for each group to preserve group difference, and so that variables measured in the TIKOL group, that were not observed in the control group (i.e., percentage of modules completed, call duration), could be included in the multiple imputation. Each multiple imputation used an unrestricted H1 model under Rubin (1987)'s missing-at-random (MAR) assumption, and 100 between-imputation (i.e., thinning) iterations. Convergence was judged to have occurred around the 5,000th iteration in both imputation models, where the proportional scale reduction (PSR) factor was close to 1.02. An ample number of iterations (i.e., 100,000) were used to for each imputation model to ensure the PSR did not prematurely approach 1.02 by chance. Variables for the same individual were recorded on one row for all time points so to preserve autocorrelational dependence. All scale variables listed in Table 2 were included in the imputation models. Results for all mixed models were pooled across imputations according to Rubin's (1987) rules.

**Table 2:** Mixed effects modeling: parent and child outcomes.

				**Adjusted means** ^**ab**^				**Test of interaction**						
				**Baseline**		**Follow-up**								
	**Measures**	**Condition**	***N***	***M***	***SE***	***M***	***SE***	β	***SE***	***t***	***p***	**95% CI**		***d***
Parent	Difficulties in ER^d^	Intervention	78	38.545	1.962	37.263	2.036	−1.701	1.646	−1.033	0.303	−4.955	1.554	−0.146
		Control	73	36.928	2.154	37.347	2.198							
	Anxiety^d^	Intervention	78	1.196	0.324	1.272	0.343	0.085	0.354	0.241	0.810	−0.614	0.785	0.043
		Control	73	1.559	0.355	1.550	0.366							
	EC beliefs^d^	Intervention	77	42.545	0.865	41.964	0.903	−0.903	0.862	−1.048	0.296	−2.608	0.801	−0.172
		Control	71	42.380	0.961	42.702	0.984							
	Empathy^d^	Intervention	77	18.545	0.505	19.900	0.528	1.055	0.519	2.032	**0.044**	0.029	2.082	0.324
		Control	72	18.598	0.557	18.897	0.572							
	ED beliefs^d^	Intervention	76	33.298	1.007	28.395	1.044	−3.987	0.919	−4.341	**0.000**	−5.805	−2.170	−0.635
		Control	70	33.079	1.119	32.163	1.141							
	EC behaviors^d^	Intervention	78	5.680	0.114	6.129	0.119	0.324	0.104	3.105	**0.002**	0.118	0.530	0.411
		Control	73	5.312	0.126	5.437	0.129							
	ED behaviors	Intervention	78	2.519	0.111	2.086	0.116	−0.412	0.107	−3.840	**0.000**	−0.624	−0.200	−0.569
		Control	73	2.091	0.123	2.070	0.126							
	Efficacy^d^	Intervention	78	56.273	1.882	50.467	1.949	−3.540	1.526	−2.320	**0.022**	−6.558	−0.523	−0.331
		Control	73	53.190	2.077	50.925	2.120							
Child	BP intensity^ce^	Intervention	78	144.065	4.893	127.622	5.021	−2.598	3.615	−0.719	0.474	−9.747	4.551	−0.091
		Control	73	131.129	5.409	117.285	5.497							
	BP problem score^cde^	Intervention	78	19.405	1.227	13.415	1.281	−1.625	1.237	−1.314	0.191	−4.071	0.820	−0.219
		Control	73	13.644	1.353	9.280	1.389							
	Anxiety	Intervention	78	4.567	0.921	2.962	0.963	−2.614	0.862	−3.031	**0.003**	−4.320	−0.908	−0.440
		Control	73	4.724	1.011	5.733	1.035							
Teacher	BP intensity	Intervention	64	92.929	7.420	90.752	7.797	11.705	7.166	1.633	0.105	−2.487	25.896	0.245
		Control	72	99.196	8.176	85.315	8.330							

^a^All results control for baseline emotion dismissing behaviors and number of support calls received.

^b^Estimated marginal means (*M*) conditioned on covariates; *SE*, Standard error; β, Unstandardised regression coefficient; *df* , degrees of freedom; *t, t*-value; *p, p*-value for *t*-statistic; 95% CI, 95% confidence interval for β; *d*, effect size for the condition^*^time interaction.

^c^Main effect of time is statistically significant at *p* < 0.05.

^d^Main effect of the covariate emotion dismissing behaviors statistically significant at *p* < 0.05.

^e^Main effect of call number statistically significant at *p* < 0.05; ER, emotion regulation; EC, emotion coaching; ED, emotion dismissing; BP, behavior problems. Bolded values are significant at <.05.

Next, covariates were determined by conducting *t*-tests and chi-square analyses that investigated baseline group differences and relationships between demographic and outcome variables. These analyses were conducted on available cases only so that these could inform potential covariates to include within the multiple imputation models. Group comparisons for demographic characteristics showed no differences between intervention and control participants, however, intervention participants reported significantly higher emotion dismissing behaviors at baseline (*M* = 57.95, *SD* = 19.02) compared with parents in the control group [*M* = 50.42, *SD* = 14.82, *t*_(149)_ = 2.699, *p* = 0.004]. Thus, baseline emotion dismissing behaviors were included as a covariate in the multiple imputation and mixed models analyses.

For the Mixed Models analyses, a baseline random intercept model (Step 1) was initially constructed for each outcome measure. As indicated by the smallest Akaike information criterion (AIC) index, best model fit for the null model was achieved using restricted maximum likelihood and a variance components covariance structure, with random intercepts and time as fixed effects (Heck et al., 2010). At Step 2, key variables (condition and time; each dummy coded 0 and 1) were added into the model, followed by covariates (parent emotion dismissing behaviors and number of support calls) at Step 3. As indicated by chi square statistics for the change in −2 Log Likelihood, adding covariates significantly improved the model (*p* < 0.01) for all outcomes of interest (Field, 2009). To investigate if engagement played a role in the intervention outcomes, parent engagement in terms of percentage of modules completed (i.e., average percentage of modules watched) and support calls (i.e., the duration of the two support calls in total) were examined as predictors of change in parent and child outcomes. The engagement variables were added as covariates in the mixed model regressions at step 4. Effect sizes were calculated using the difference between the estimated means of the slopes (unstandardized *b*-value) of the two groups (intervention and control over time) divided by the baseline standard deviation of raw scores. Effect sizes (*d*) of 0.20 are small, 0.50 medium and >0.8 large (Cohen, 1988).

## Results

### Intervention outcomes

Table 2 presents intervention outcomes, including statistics for the interaction between time and condition which, when significant, reflect a difference in slopes for the two groups (i.e., change varies depending on condition). Main effects for time are only reported in text when the interaction between time and condition was not significant.

At 9-months post baseline (~6 months after TIKOL), intervention parents reported significantly greater empathy and emotion coaching *behaviors* when compared with control parents (small effect size). Intervention parents also reported significantly greater reductions in emotion dismissing *beliefs* and *behaviors* (medium effect size), compared with control parents. Further, intervention parents reported greater parenting efficacy and satisfaction on the PSOC (small effect size) compared to control parents. The interaction between time and condition did not reach significance for parent-reported difficulties in emotion regulation and emotion coaching beliefs, with a non-significant main effect of time indicating no change on these variables. There were also no significant changes in parent reported anxiety on the DASS. Baseline emotion dismissing behaviors (covariate) was a significant predictor for all parent-reported parent outcomes and suggested that parents who reported higher levels of emotion dismissing at baseline were less likely to report improvements on all outcomes at 6 months follow-up.

For parent-reported child outcomes, interactions between time and condition indicated significantly greater reductions in parent-reported child anxiety on the SCAS compared to waitlist control participants (small effect size). Both intervention and control conditions showed changes on the ECBI intensity (**β** **=** 16.443, *SE* = 2.475, *t* = 6.664, *p* < 0.001) and problem scores (**β** **=** 5.990, *SE* = 0.849, *t* = 7.059, *p* < 0.001), from baseline to follow-up, as indicated by a significant main effect of time combined with a non-significant interaction between time and condition for ECBI intensity (*p* = 0.474) and ECBI problem scores (*p* = 0.191; see Table 2). However, there was a significant main effect of time for call number (covariate), which indicated that parents who received more support calls were more likely to report reductions in behavior intensity (**β** = −9.647, *SE* = 4.744, *t* = −2.034, *p* = 0.044) and problem scores (**β** = −3.670, S*E* = 1.165, *t* = −3.150, *p* = 0.002). There were no changes for teacher reported child behavior problems, as indicated by non-significant main effects for time and time and condition interaction effects.

To investigate if engagement played a role in the intervention outcomes, parent engagement measured by percentage of modules completed (i.e., the average percentage of the modules watched) and support calls (i.e., the duration of the two support calls in total) were examined as predictors of change for parent and child outcomes. The engagement variables were added as covariates in the mixed model regressions at step 4. Based on the median AIC across all 100 imputations, model fit did not significantly improve when module watching or call duration were added to any of the outcomes, however, call duration significantly predicted parent anxiety (β **=** 0.017, *SE* = 0.008, *t* = 2.115, *p* < 0.036), although the effect size for this was very small at 0.008. The addition of the two engagement variables in step 4 resulted in the main effect for call number becoming non-significant for parent anxiety (β **=** −0.255, *SE* = 0.405, *t* = −6.29, *p* < 0.530). The addition of the engagement variables did not alter the interaction effects between time and condition for any outcome reported in Table 2.

All analyses were repeated using the ITT data set. Results did not differ, with the exception of parent reported empathy, where the interaction effect became statistically non-significant (**β=** 0.953, *SE* = 0.531, *t* = 1.795, *p* < 0.073, *d* = 0.287), although a small effect size remained. Instead a significant main effect of time (β = −1.354, SE = 0.357, *t* = −3.796, *p* = 0.000) and call number (β = 0.020, SE = 0.478, *t* = 0.042, *p* = 0.966) were found, indicating that all parents reported changes over time, but that parents with a greater number of calls reported larger changes in empathy.

## Discussion

This randomized control trial evaluated whether an online emotion-focused parenting program, *Tuning in to Kids OnLine* (TIKOL), was efficacious in improving parent emotion socialization and reducing child internalizing and externalizing problems in a sample of parents of 4–10 year old children with challenging behaviors. Additionally, the study aimed to explore if engagement in module completion and/or phone support calls could enhance program outcomes. Parents in the TIKOL condition reported significantly greater improvements in empathy, emotion coaching behaviors and parent self-efficacy and significantly greater reductions in parent emotion dismissing beliefs and behaviors following the program, when compared to control participants. While all parents reported changes in child behavior problems, changes were greater for parents who received more phone support calls. Additionally, intervention parents reported greater reductions in child anxiety at 6 months follow up. Parent self-reported anxiety, difficulties in emotion regulation and emotion coaching beliefs did not change. While the number of calls intervention parents received impacted parent-reported anxiety and child behavior problems in positive ways, the percentage of module completion and duration of support calls were not associated with outcomes. Teachers did not report changes in children's behavior problems. These findings are partially consistent with other TIK studies where parents received the program via group or one-to-one delivery (Havighurst et al., 2010, 2013, 2015; Mastromanno et al., 2021) and show online delivery of TIK may offer an effective way of improving parent emotion socialization and anxiety in children with challenging behaviors. This study also highlights the importance of online dosage, with support call assisted delivery resulting in greater reductions in behavior problems.

### Program efficacy

Our first aim was to examine program efficacy on parent and child outcomes. Parent emotion socialization includes attitudes or beliefs about emotions as well as how parents respond to emotions when they occur. TIKOL appears to have had an impact on parents' self-reported emotion dismissing beliefs and practices surrounding their responsiveness to emotions in their children, and also led to significant changes in parent-reported emotion coaching practices. Many studies have found that parents often hold beliefs that emotions should be avoided, dampened or managed privately—rather than engaging with their children when emotions are experienced and valuing them as opportunities to connect and emotion coach (Dunsmore et al., 2009; Parker et al., 2012; Gross and Cassidy, 2019). Changing dismissing beliefs about emotions, that are often held with conviction, and then learning new parenting skills, are key components of interventions (Gutentag et al., 2017; Ford and Gross, 2019). Although outcomes for TIKOL were slightly smaller effect sizes when compared with community sample trials, they were consistent with other TIK studies when the program was delivered one-to-one with children aged 4–11 with challenging behaviors (Mastromanno et al., 2021), in group delivery with children 4–5 years attending a clinical service with behavior problems (Havighurst et al., 2013) and in group delivery with 4–10 year old children with emerging conduct problems (Havighurst et al., 2015) where effect sizes with emotion dismissing parenting beliefs and practices were medium to large. Previous TIK studies have also demonstrated significant improvements in parent emotion socialization with observation measures, verifying that parent self-reports are likely indicative of change and not due to expectancy bias (Havighurst et al., 2010, 2013, 2015). The current study with TIKOL did not include observation outcomes, however, with similar program content as in other delivery variants of TIK, parent-reported changes are likely indicators of emotion socialization change in practice. The current study suggests that it is possible to shift parent's dismissing beliefs about responding to emotions and improve emotion socialization practices using TIKOL. Given that the original TIK program for parents with greater challenges involves an 8 × 2 h session delivery, this is a significant cost saving with implications for prevention and intervention.

As parents also reported greater efficacy in their parenting, this suggests that even with an online parenting program, the benefits from the intervention are not just in terms of emotion socialization skills but also increases in confidence in parenting. Previous research has indicated that when parents feel competent, they are likely to use more effective parenting, including being more sensitive, responsive and warm (Bloomfield et al., 2005; Wittkowski et al., 2016). Additionally, parent self-efficacy has been found to act as a buffer against stress and adverse risk factors such as disadvantaged socioeconomic situations (Williams, 2020).

Parent emotion regulation difficulties and anxiety were not reported to change in intervention or control participants. This light dose of the TIKOL program included some information about self-care and emotion regulation for parents, however, a more substantial component addressing this topic or therapist assisted support may be required to change this aspect of parent functioning.

The convenience of being able to access parent education and support online while building skills and confidence, overcomes several of the barriers encountered in face-to-face programs. Barriers include time constraints and scheduling conflicts related to attending face-to-face sessions and concerns related to stigma and lack of anonymity (Sim et al., 2022). The addition of support calls has also been found to enhance engagement in online programs (Collins et al., 2019; Hansen et al., 2019). In the current study, participants had at least one phone call with a greater number of calls being associated with greater changes in parent-reported child behavior problems and longer calls being associated with greater reduction for parent anxiety. This finding is consistent with other online programs where support calls enhance outcomes (e.g., Day and Sanders, 2018; Sim et al., 2022).

Following TIKOL, there were also significant reductions in parent-reported child behavior problems (intensity and problem scores on the ECBI) and anxiety (using the SCAS), with small effect sizes. While the control parents also reported reductions in child behavior problems, greater changes were reported by parents who received more support calls reflecting a more substantial dose of the program. This is partially consistent with previous studies of TIK that have resulted in parent-reported reductions in children's behavior problems (Havighurst et al., 2010, 2013, 2015), however, it highlights the importance of support call assisted delivery of TIKOL to reduce behavior problems. It may be that parents required more practice in emotion coaching (e.g., via therapist guidance or role plays), an integral part of other delivery modalities of the TIK program in group and one-to-one format, which is missing in the online delivery. In the support calls, parents were able to discuss specific situations where their child engaged in challenging behavior and were guided via role play in how to use emotion coaching.

In the current study, there were no significant reductions on teacher reported behavior, however, floor effects on the SESBI at baseline suggest teachers did not see behavior as a problem for children. Given our sample included those with internalizing and externalizing behaviors, these difficulties may not have been evident in the school context. Further, with follow-up evaluation occurring during COVID lockdown, data for many participants was not possible to collect due to research being stopped by the relevant government department.

A novel finding of this study was the impact of TIKOL on child anxiety. In the preschool and primary school years, challenging behaviors in children often include comorbidity of internalizing (anxiety, withdrawal) and externalizing (oppositional, aggressive) behaviors (Willner et al., 2016). Transdiagnostic interventions such as TIKOL, that address the underlying influences (such as parent emotion socialization) and impact on developmental processes (such as children's emotion regulation), will have the added benefit of impacting several aspects of functioning (McEvoy et al., 2009; Dalgleish et al., 2020). The current study demonstrated that the same intervention addressed the emotional and behavioral difficulties that parents were experiencing with their children. Many other studies have found that online programs have positive impacts on reducing child behavior problems and anxiety (Morgan et al., 2017; Yap et al., 2018), however this the first study to demonstrate this using an online program based on emotion socialization theory (rather than behavioral theory).

### Program engagement

The second study aim was to examine how engagement impacted parent and child outcomes. This was measured by examining the amount of the program parents watched (percentage of module completion) and their use of support calls (duration and number of calls). There were 10 modules consisting of 5–15 min videos with additional practice tasks. Module watching was high (78% watched at least 60%) indicating that parents were engaged, but analyses found that greater module completion was not associated with greater improvements on outcomes.

In terms of engagement in the phone calls, call duration was related to greater reductions in parent anxiety but was not a significant factor contributing to decreased behavior problems. However, the number of calls parents received was significant, especially for helping reduce child behavior problems. Phone calls were used in TIKOL to engage parents and to help them apply the online content to their target child. Two calls were offered to each parent, with an average of 29 min duration and involved working through at least one example of an emotionally challenging situation with their child. In some cases the calls were able to identify when parents had incorrectly jumped to conclusions about how to use the content—and assisted with correction and support. It may be that parents who received more than one call were able to try out and then apply the skills and check in one more time during the support call to refine skills, similar to when parents learn the skills in the group version of TIK. A follow-up call may have helped to iron out any other misunderstandings. For example, some parents appeared to be using the five steps of emotion coaching in quick succession (e.g., noting their child's downcast face, coming in close, saying, “That's hard. I see you are sad. What can we do?”) rather than allowing time after steps 3 and 4 (empathy and naming emotions) where the child's emotion might first reduce in intensity before step 5 (problem solving/limit setting) was used. Phone calls aimed to assist parents with the nuances of emotion coaching to develop the new skills (Orji et al., 2012; Finan et al., 2018). Indeed, skill mastery is consistently recognized as an integral component of behavior change (Bandura, 1994; Orji et al., 2012; Finan et al., 2018), with phone calls making a significant contribution with TIKOL.

The calls were also important for parent anxiety, perhaps because they enabled phone workers to guide parents in how they were coping, prompting self-care and emotion regulation to assist in their wellbeing. Additionally, phone support workers were trained to model emotion coaching with parents, providing empathy and support before assisting parents with how to use the skills with their child. This supported parents' wellbeing, fostered emotion acceptance, and modeled how to emotion coach. Other studies have also found that support for parents is integral to reducing their stress helping them to be better able to meet their child's needs (Breitenstein et al., 2014; Barlow et al., 2016; Wittkowski et al., 2016).

### Limitations

This study used parent-reported outcomes only and did not independently verify changes by conducting observations of parenting or children's functioning. Parent reports can be subject to expectancy bias, therefore limiting reliance on this type of data is important in establishing efficacy. Prior studies of TIK have included observation measures and direct child emotional competence assessments, where parents were observed to have improved emotion socialization with medium to large effect sizes and children improved with small to medium effect sizes (Havighurst et al., 2010, 2013, 2015). Reassuringly, in these same studies, parent-reported changes had similar (or slightly larger) effect sizes to those in the current study. Additionally, independent assessment of child behavior and anxiety could strengthen conclusions regarding the reported changes by parents. Unfortunately, teacher follow-up data was minimal due to COVID lockdowns preventing research being conducted in schools. Finally, because the study was conducted prior to and during the COVID pandemic, examining whether there were cohort effects due to these external challenges would have been interesting. However, examination of COVID as a covariate did not add to the model, possibly due to a small sample or because it did not impact outcomes. Longer follow-up would also have been beneficial to see if changes in parent and child functioning were sustained.

## Conclusion

This is the first study of an online self-paced, emotion socialization parenting program—*Tuning in to Kids OnLine* (TIKOL), evaluated using a randomized control design with 6-month follow up. The program was efficacious in reducing emotion dismissive parenting, while increasing empathy and emotion coaching—key aspects of healthy emotion socialization. Greater parental efficacy and confidence were found, and parents also reported reductions in their children's behavior problems and anxiety. These are similar changes found in one-to-one or group delivery of TIK but with slightly smaller effect sizes. TIKOL has the potential to overcome barriers to parenting program attendance including cost, accessibility and parental fears/guilt, but without benefits that a group can provide including social support, normalizing of parenting challenges or opportunities to practice skills. Further evaluation with a sample from more diverse socio-economic backgrounds and using independent observations of parenting and child functioning would strengthen support for the parent reported findings. Phone support calls were found to enhance engagement, resulting in greater reductions in parental anxiety and improvements in children's behavioral functioning.

## Data Availability

The datasets presented in this article are not readily available because participants have not given informed consent for their data to be publicly accessible. Requests to access the datasets should be directed to the corresponding author SH, sophie.h@unimelb.edu.au.

## References

[B1] Aghaie Meybodi F. Mohammadkhani P. Pourshahbaz A. Dolatshahi B. Havighurst S. S. (2019). Improving parent emotion socialization practices: piloting tuning in to kids in Iran for children with disruptive behavior problems. Fam. Relat. 68, 596–607. 10.1111/fare.12387

[B2] Arendt K. Hougaard E. Thastum M. (2014). Psychometric properties of the child and parent versions of Spence children's anxiety scale in a Danish community and clinical sample. J. Anxiety Disord. 28, 947–956. 10.1016/j.janxdis.2014.09.021 25445085

[B3] Australian Bureau of Statistics (2019). Personal Income in Australia (2014-15-to-2018-19). Australian Bureau of Statistics. Available at: https://www.abs.gov.au/statistics/labour/earnings-and-working-conditions/personal-income-australia/2014-15-2018-19 (accessed December 10, 2023).

[B4] Bandura A. (1994). Self-Efficacy in Encyclopedia of Human Behavior. New York, NY: Academic Press.

[B5] Barlow J. Bergman H. Kornør H. Wei Y. Bennett C. (2016). Group-based parent training programmes for improving emotional and behavioural adjustment in young children. Cochr. Database Syst. Rev. 2016:CD003680. 10.1002/14651858.CD003680.pub3 27478983 PMC6797064

[B6] Baumel A. Pawar A. Kane J. M. Correll C. U. (2016). Digital parent training for children with disruptive behaviors: systematic review and meta-analysis of randomized trials. J. Child Adolesc. Psychopharmacol. 26, 740–749. 10.1089/cap.2016.0048 27286325

[B7] Baumel A. Pawar A. Mathur N. Kane J. M. Correll C. U. (2017). Technology-assisted parent training programs for children and adolescents with disruptive behaviors: a systematic review. J. Clin. Psychiatry 78, e957–e969. 10.4088/JCP.16r11063 28493653

[B8] Bjureberg J. Ljótsson B. Tull M. T. Hedman E. Sahlin H. Lundh L.-G.. (2016). Development and validation of a brief version of the difficulties in emotion regulation scale: the DERS-16. J. Psychopathol. Behav. Assess. 38, 284–296. 10.1007/s10862-015-9514-x 27239096 PMC4882111

[B9] Bloomfield L. Kendall S. Applin L. Attarzadeh V. Dearnley K. Edwards L.. (2005). A qualitative study exploring the experiences and views of mothers, health visitors and family support centre workers on the challenges and difficulties of parenting. Health Soc. Care Commun. 13, 46–55. 10.1111/j.1365-2524.2005.00527.x 15717906

[B10] Bølstad E. Havighurst S. S. Tamnes C. K. Nygaard E. Bjørk R. F. Stavrinou M.. (2021). A pilot study of a parent emotion socialization intervention: impact on parent behavior, child self-regulation, and adjustment. Front. Psychol. 12:730278. 10.3389/fpsyg.2021.730278 34721193 PMC8554311

[B11] Breitenstein S. M. Fogg L. Ocampo E. V. Acosta D. I. Gross D. (2016). Parent use and efficacy of a self-administered, tablet-based parent training intervention: a randomized controlled trial. JMIR mHealth uHealth 4:e36. 10.2196/mhealth.5202 27098111 PMC4867750

[B12] Breitenstein S. M. Gross D. Christophersen R. (2014). Digital delivery methods of parenting training interventions: a systematic review. Worldviews Evid. Based Nurs. 11, 168–176. 10.1111/wvn.12040 24842341

[B13] Burkhardt S. C. A. Röösli P. Müller X. (2024). The Tuning in to Kids parenting program delivered online improves emotion socialization and child behavior in a first randomized controlled trial. Sci. Rep. 14:4979. 10.1038/s41598-024-55689-z 38424200 PMC10904363

[B14] Chan R. F. Y. Qiu C. Shum K. K. (2021). Tuning in to kids: a randomized controlled trial of an emotion coaching parenting program for Chinese parents in Hong Kong. Dev. Psychol. 57, 1796–1809. 10.1037/dev0001258 34914446

[B15] Cohen J. (1988). Statistical Power Analysis for the Behavioral Sciences. L. Erlbaum Associates. Available at: https://books.google.com.au/books?id=gA04ngAACAAJ (accessed December 10, 2023).

[B16] Collins D. A. J. Tully L. A. Piotrowska P. J. Hawes D. J. Dadds M. R. (2019). Perspectives on ParentWorks: learnings from the development and national roll-out of a self-directed online parenting intervention. Int. Interven. 15, 52–59. 10.1016/j.invent.2018.12.002 30656140 PMC6329694

[B17] Cugelman B. Thelwall M. Dawes P. (2011). Online interventions for social marketing health behavior change campaigns: a meta-analysis of psychological architectures and adherence factors. J. Med. Int. Res. 13:e17. 10.2196/jmir.1367 21320854 PMC3221338

[B18] Cuijpers P. van Straten A. Warmerdam L. van Rooy M. J. (2010). Recruiting participants for interventions to prevent the onset of depressive disorders: possibile ways to increase participation rates. BMC Health Serv. Res. 10:181. 10.1186/1472-6963-10-181 20579332 PMC2907376

[B19] Dalgleish T. Black M. Johnston D. Bevan A. (2020). Transdiagnostic approaches to mental health problems: current status and future directions. J. Consult. Clin. Psychol. 88, 179–195. 10.1037/ccp0000482 32068421 PMC7027356

[B20] Day J. J. Sanders M. R. (2018). Do parents benefit from help when completing a self-guided parenting program online? A randomized controlled trial comparing Triple P Online with and without telephone support. Behav. Therapy 49, 1020–1038. 10.1016/j.beth.2018.03.002 30316482

[B21] De Graaf I. Speetjens P. Smit F. De Wolff M. Tavecchio L. (2008). Effectiveness of the Triple P Positive Parenting Program on behavioral problems in children. Behav. Modif. 32, 714–735. 10.1177/0145445508317134 18475003

[B22] Dunsmore J. C. Her P. Halberstadt A. G. Perez-Rivera M. B. (2009). parents' beliefs about emotions and children's recognition of parents' emotions. J. Nonverb. Behav. 33, 121–140. 10.1007/s10919-008-0066-6 20160992 PMC2755301

[B23] Edrissi F. Havighurst S. S. Aghebati A. Habibi M. Arani A. M. (2019). A pilot study of the Tuning in to Kids Parenting Program in iran for reducing preschool children's anxiety. J. Child Fam. Stud. 28, 1695–1702. 10.1007/s10826-019-01400-0

[B24] Eisenberg N. Cumberland A. Spinrad T. L. (1998). Parental socialization of emotion. Psychol. Inq. 9, 241–273. 10.1207/s15327965pli0904_1 16865170 PMC1513625

[B25] Enebrink P. Högström J. Forster M. Ghaderi A. (2012). Internet-based parent management training: a randomized controlled study. Behav. Res. Ther. 50, 240–249. 10.1016/j.brat.2012.01.006 22398153

[B26] Eyberg S. M. Pincus D. (1999). Eyberg Child Behavior Inventory and Sutter–Eyberg Student Behavior Inventory: Professional Manual. Odessa, FL: Psychological Assessment Resources.

[B27] Fabes R. Eisenberg N. Bernzweig J. (1990). The Coping With Children's Negative Emotions Scale: Description and Scoring. Unpublished scale, Department of Family Resources and Human Development, Temple. Arizona: Arizona State University.

[B28] Fabes R. A. Poulin R. E. Eisenberg N. Madden-Derdich D. A. (2002). The coping with children's negative emotions scale (CCNES): Psychometric properties and relations with children's emotional competence. Marr. Fam. Rev. 34, 285–310. 10.1300/J002v34n03_05

[B29] Field A. (2009). Discovering Statistics Using SPSS, 3rd Edn. London: Sage.

[B30] Finan S. J. Swierzbiolek B. Priest N. Warren N. Yap M. (2018). Parental engagement in preventive parenting programs for child mental health: a systematic review of predictors and strategies to increase engagement. PeerJ 6:e4676. 10.7717/peerj.4676 29719737 PMC5926551

[B31] Flujas-Contreras J. M. García-Palacios A. Gómez I. (2019). Technology-based parenting interventions for children's physical and psychological health: a systematic review and meta-analysis. Psychol. Med. 49, 1787–1798. 10.1017/S0033291719000692 30977462

[B32] Ford B. Q. Gross J. J. (2019). Why beliefs about emotion matter: an emotion-regulation perspective. Curr. Dir. Psychol. Sci. 28, 74–81. 10.1177/0963721418806697

[B33] Gibaud-Wallston J. Wandersman L. P. (1978). Development and utility of the Parenting Sense of Competence Scale. Paper presented at the annual meeting of the American Psychological Association, Toronto.

[B34] Gottman J. M. DeClaire J. (1997). Raising an Emotionally Intelligent Child: The Heart of Parenting. New York, NY: Simon & Schuster.

[B35] Gottman J. M. Katz L. F. Hooven C. (1996). Parental meta-emotion philosophy and the emotional life of families: theoretical models and preliminary data. J. Fam. Psychol. 10, 243–268. 10.1037/0893-3200.10.3.243

[B36] Gross J. T. Cassidy J. (2019). Expressive suppression of negative emotions in children and adolescents: theory, data, and a guide for future research. Dev. Psychol. 55, 1938–1950. 10.1037/dev0000722 31464496

[B37] Gutentag T. Halperin E. Porat R. Bigman Y. E. Tamir M. (2017). Successful emotion regulation requires both conviction and skill: beliefs about the controllability of emotions, reappraisal, and regulation success. Cogn. Emot. 31, 1225–1233. 10.1080/02699931.2016.1213704 27494261

[B38] Hallion L. S. Steinman S. A. Tolin D. F. Diefenbach G. J. (2018). Psychometric Properties of the Difficulties in Emotion Regulation Scale (DERS) and its short forms in adults with emotional disorders. Front. Psychol. 9:539. 10.3389/fpsyg.2018.00539 29725312 PMC5917244

[B39] Hansen A. Broomfield G. Yap M. B. H. (2019). A systematic review of technology-assisted parenting programs for mental health problems in youth aged 0–18 years: applicability to underserved Australian communities. Aust. J. Psychol. 71, 433–462. 10.1111/ajpy.12250

[B40] Havighurst S. S. Chainey C. Doyle F. L. Higgins D. J. Mathews B. Mazzucchelli T. G.. (2022). A review of Australian Government funding of parenting intervention research. Aust. N. Z. J. Public Health 46, 262–268. 10.1111/1753-6405.13235 35436026

[B41] Havighurst S. S. Duncombe M. Frankling E. Holland K. Kehoe C. Stargatt R. (2015). An emotion-focused early intervention for children with emerging conduct problems. J. Abnorm. Child Psychol. 43, 749–760. 10.1007/s10802-014-9944-z 25249470

[B42] Havighurst S. S. Harley A. Prior M. (2004). Building preschool children's emotional competence: a parenting program. Early Educ. Dev. 15, 423–448. 10.1207/s15566935eed1504_5

[B43] Havighurst S. S. Murphy J. L. Kehoe C. E. (2021). Trauma-focused Tuning in to Kids: evaluation in a clinical service. Children 8:1038. 10.3390/children8111038 34828751 PMC8620103

[B44] Havighurst S. S. Radovini A. Hao B. Kehoe C. E. (2020). Emotion-focused parenting interventions for prevention and treatment of child and adolescent mental health problems: a review of recent literature. Curr. Opin. Psychiatry 33, 586–601. 10.1097/YCO.0000000000000647 32858599

[B45] Havighurst S. S. Wilson K. R. Harley A. E. Kehoe C. Efron D. Prior M. R. (2013). “Tuning into Kids”: reducing young children's behavior problems using an emotion coaching parenting program. Child Psychiatry Hum. Dev. 44, 247–264. 10.1007/s10578-012-0322-1 22820873

[B46] Havighurst S. S. Wilson K. R. Harley A. E. Prior M. R. Kehoe C. (2010). Tuning in to Kids: improving emotion socialization practices in parents of preschool children – findings from a community trial. J. Child Psychol. Psychiatry 51, 1342–1350. 10.1111/j.1469-7610.2010.02303.x 20735794

[B47] Heck R. H. Thomas S. L. Tabata L. N. (2010). Multilevel and Longitudinal Modeling With IBM SPSS: Quantitative Methodology Series. New York, NY: Rutledge.

[B48] Jones S. H. Jovanoska J. Calam R. Wainwright L. D. Vincent H. Asar O.. (2017). Web-based integrated bipolar parenting intervention for parents with bipolar disorder: a randomised controlled pilot trial. J. Child Psychol. Psychiatry 58, 1033–1041. 10.1111/jcpp.12745 28512921 PMC5573909

[B49] Lagacé-Séguin D. G. Coplan R. J. (2005). Maternal emotional styles and child social adjustment: assessment, correlates, outcomes and goodness of fit in early childhood. Soc. Dev. 14, 613–636. 10.1111/j.1467-9507.2005.00320.x

[B50] Little R. J. A. (1988). A test of missing completely at random for multivariate data with missing values. J. Am. Stat. Assoc. 83, 1198–1202. 10.1080/01621459.1988.10478722

[B51] Lovibond P. F. Lovibond S. H. (1995). The structure of negative emotional states: comparison of the Depression Anxiety Stress Scales (DASS) with the Beck Depression and Anxiety Inventories. Behav. Res. Therapy 33, 335–343. 10.1016/0005-7967(94)00075-U 7726811

[B52] Mastromanno B. K. Kehoe C. E. Wood C. E. Havighurst S. S. (2021). Tuning in to Kids: clinical case studies from one-to-one delivery. Clin. Case Stud. 20, 267–282. 10.1177/1534650120983909

[B53] McEvoy P. M. Nathan P. Norton P. J. (2009). Efficacy of transdiagnostic treatments: a review of published outcome studies and future research directions. J. Cogn. Psychother. 23, 20–33. 10.1891/0889-8391.23.1.2038151800

[B54] Means B. Toyama Y. Murphy R. Bakia M. Jones K. Planning E. (2010). Evaluation of Evidence-Based Practices in Online Learning: A Meta-Analysis and Review of Online Learning Studies. Available at: http://lst-iiep.iiep-unesco.org/cgi-bin/wwwi32.exe/[in=epidoc1.in]/?t2000=027003/115.

[B55] Metzler C. W. Sanders M. R. Rusby J. C. Crowley R. N. (2012). Using consumer preference information to increase the reach and impact of media-based parenting interventions in a public health approach to parenting support. Behav. Ther. 43, 257–270. 10.1016/j.beth.2011.05.004 22440064 PMC3442606

[B56] Morgan A. J. Rapee R. M. Salim A. Goharpey N. Tamir E. McLellan L. F.. (2017). Internet-delivered parenting program for prevention and early intervention of anxiety problems in young children: randomized controlled trial. J. Am. Acad. Child Adolesc. Psychiatry 56 417–425.e411. 10.1016/j.jaac.2017.02.010 28433091

[B57] Muthén L. K. Muthén B. O. (1998–2017). Mplus User's Guide, 8th Edn. Los Angeles, CA: Muthén & Muthén.

[B58] Nauta M. H. Scholing A. Rapee R. M. Abbott M. Spence S. H. Waters A. (2004). A parent-report measure of children's anxiety: psychometric properties and comparison with child-report in a clinic and normal sample. Behav. Res. Therapy 42, 813–839. 10.1016/S0005-7967(03)00200-6 15149901

[B59] Nieuwboer C. C. Fukkink R. G. Hermanns J. M. (2013). Peer and professional parenting support on the Internet: a systematic review. Cyberpsychol. Behav. Soc. Netw. 16, 518–528. 10.1089/cyber.2012.0547 23659725

[B60] Nowak C. Heinrichs N. (2008). A comprehensive meta-analysis of Triple P-Positive Parenting Program using hierarchical linear modeling: effectiveness and moderating variables. Clin. Child Fam. Psychol. Rev. 11:114. 10.1007/s10567-008-0033-0 18509758

[B61] Orji R. Vassileva J. Mandryk R. (2012). Towards an effective health interventions design: an extension of the health belief model. J. Public Health Inform. 4:4321. 10.5210/ojphi.v4i3.4321 23569653 PMC3615835

[B62] Owens P. L. Hoagwood K. Horwitz S. M. Leaf P. J. Poduska J. M. Kellam S. G.. (2002). Barriers to children's mental health services. J. Am. Acad. Child Adolesc. Psychiatry 41, 731–738. 10.1097/00004583-200206000-00013 12049448

[B63] Parker A. E. Halberstadt A. G. Dunsmore J. C. Townley G. Bryant A. Jr. Thompson J. A.. (2012). Emotions are a window into one's heart”: a qualitative analysis of parental beliefs about children's emotions across three ethnic groups. Monogr. Soc. Res. Child Dev. 77, 1–136. 10.1037/t27811-000 22905794

[B64] Prinz R. J. Sanders M. R. (2007). Adopting a population-level approach to parenting and family support interventions. Clin. Psychol. Rev. 27, 739–749. 10.1016/j.cpr.2007.01.005 17336435

[B65] Querido J. G. Eyberg S. M. (2003). Psychometric properties of the sutter-eyberg student behavior inventory-revised with preschool children. Behav. Ther. 34, 1–15. 10.1016/S0005-7894(03)80018-714994955

[B66] Ramme R. (2008). S*pence Children's Anxiety Scale: An Overview of Psychometric Findings*. Available at: https://www.scaswebsite.com/wp-content/uploads/2021/07/Ramme-SCAS-Psychomet-evidence.pdf (Retrieved January 29, 2019).

[B67] Richards D. Richardson T. (2012). Computer-based psychological treatments for depression: a systematic review and meta-analysis. Clin. Psychol. Rev. 32, 329–342. 10.1016/j.cpr.2012.02.004 22466510

[B68] Rogers H. Matthews J. (2004). The parenting sense of competence scale: Investigation of the factor structure, reliability, and validity for an Australian sample. Aust. Psychol. 39, 88–96. 10.1080/00050060410001660380

[B69] Rubin D. B. (1987). Multiple Imputation for Nonresponse in Surveys. New York, NY: Wiley.

[B70] Sanders M. R. Baker S. Turner K. M. (2012). A randomized controlled trial evaluating the efficacy of Triple P Online with parents of children with early-onset conduct problems. Behav. Res. Ther. 50, 675–684. 10.1016/j.brat.2012.07.004 22982082

[B71] Schulz K. F. Altman D. G. Moher D. (2010). CONSORT 2010 statement: Updated guidelines for reporting parallel group randomised trials. Br. Med. J. 340*:*c332. 10.1136/bmj.c332 20332509 PMC2844940

[B72] Scott S. Dadds M. R. (2009). Practitioner review: when parent training doesn't work: theory-driven clinical strategies. J. Child Psychol. Psychiatry 50, 1441–1450. 10.1111/j.1469-7610.2009.02161.x 19754503

[B73] Sim W. H. Jorm A. F. Yap M. B. H. (2022). The role of parent engagement in a web-based preventive parenting intervention for child mental health in predicting parenting, parent and child outcomes. Int. J. Environ. Res. Public Health 19:2191. 10.3390/ijerph19042191 35206394 PMC8871768

[B74] Sourander A. McGrath P. J. Ristkari T. Cunningham C. Huttunen J. Lingley-Pottie P.. (2016). Internet-assisted parent training intervention for disruptive behavior in 4-year-old children: a randomized clinical trial. JAMA Psychiatry 73, 378–387. 10.1001/jamapsychiatry.2015.3411 26913614

[B75] Spoth R. Redmond C. (2000). Research on family engagement in preventive interventions: toward improved use of scientific findings in primary prevention practice. J. Prim. Prev. 21, 267–284. 10.1023/A:1007039421026

[B76] Thongseiratch T. Leijten P. Melendez-Torres G. J. (2020). Online parent programs for children's behavioral problems: a meta-analytic review. Eur. Child Adolesc. Psychiatry 29, 1555–1568. 10.1007/s00787-020-01472-0 31925545

[B77] Urbaniak G. C. Plous S. (2013). Research Randomizer (Version 4.0) [Computer software]. Available at: http://www.randomizer.org/ (accessed December 10, 2023).

[B78] Wetterborg D. Enebrink P. Lönn Rhodin K. Forster M. Risto E. Dahlström J.. (2019). A pilot randomized controlled trial of Internet-delivered parent training for parents of teenagers. J. Fam. Psychol. 33, 764–774. 10.1037/fam0000541 31204818

[B79] Whiteside S. P. Brown A. M. (2008). Exploring the utility of the Spence Children's Anxiety Scales parent- and child-report forms in a North American sample. J. Anxiety Disord. 22, 1440–1446. 10.1016/j.janxdis.2008.02.006 18395408

[B80] Williams C. (2020). Parental Self-Efficacy and Parenting Through Adversity. 10.5772/intechopen.91735

[B81] Willner C. J. Gatzke-Kopp L. M. Bray B. C. (2016). The dynamics of internalizing and externalizing comorbidity across the early school years. Dev. Psychopathol. 28, 1033–1052. 10.1017/S0954579416000687 27739391 PMC5319409

[B82] Wilson K. R. Havighurst S. S. Harley A. E. (2012). Tuning in to Kids: an effectiveness trial of a parenting program targeting emotion socialization of preschoolers. J. Fam. Psychol. 26, 56–65. 10.1037/a0026480 22182335

[B83] Wilson K. R. Havighurst S. S. Harley A. E. (2014). Dads Tuning in to Tids: piloting a new parenting program targeting fathers' emotion coaching skills. J. Community Psychol. 42, 162–168. 10.1002/jcop.21601

[B84] Wittkowski A. Dowling H. Smith D. M. (2016). Does engaging in a group-based intervention increase parental self-efficacy in parents of preschool children? A systematic review of the current literature. J. Child Fam. Stud. 25, 3173–3191. 10.1007/s10826-016-0464-z 27795657 PMC5061830

[B85] Yap M. B. H. Mahtani S. Rapee R. M. Nicolas C. Lawrence K. A. Mackinnon A.. (2018). A tailored web-based intervention to improve parenting risk and protective factors for adolescent depression and anxiety problems: postintervention findings from a randomized controlled trial. J. Med. Int. Res. 20:e17. 10.2196/jmir.9139 29351895 PMC5797292

